# New MRI contrast agents based on silicon nanotubes loaded with superparamagnetic iron oxide nanoparticles

**DOI:** 10.1098/rsos.180697

**Published:** 2018-08-01

**Authors:** Roberto Gonzalez-Rodriguez, Petra Granitzer, Klemens Rumpf, Jeffery L. Coffer

**Affiliations:** 1 Department of Chemistry, Texas Christian University, Fort Worth, TX 76129 USA; 2 Institute of Physics, Karl-Franzens-University Graz, Universitaetsplatz 5, 8010 Graz, Austria

**Keywords:** silicon nanotubes, iron oxide, MRI, contrast agent

## Abstract

This article describes the preparation and fundamental properties of a new possible material as a magnetic resonance imaging contrast agent based on the incorporation of preformed iron oxide (Fe_3_O_4_) nanocrystals into hollow silicon nanotubes (Si NTs). Specifically, superparamagnetic Fe_3_O_4_ nanoparticles of two different average sizes (5 nm and 8 nm) were loaded into Si NTs of two different shell thicknesses (40 nm and 70 nm). To achieve proper aqueous solubility, the NTs were functionalized with an outer polyethylene glycol-diacid (600) moiety via an aminopropyl linkage. Relaxometry parameters *r*_1_ and *r*_2_ were measured, with the corresponding *r*_2_/*r*_1_ ratios in phosphate buffered saline confirming the expected negative contrast agent behaviour for these materials. For a given nanocrystal size, the observed *r*_2_ values are found to be inversely proportional to NT wall thickness, thereby demonstrating the role of nanostructured silicon template on associated relaxometry properties.

## 1. Introduction

Superparamagnetic iron oxide nanoparticles (Fe_3_O_4_ NPs) are the focus of extensive attention in areas such as catalysis [1], as well as biomedical applications such as cell labelling [2], biosensing [3], drug delivery [4], hyperthermia [5] and magnetic resonance imaging (MRI) [6]. In addition to their size-selective synthesis and useful fundamental magnetic properties, Fe_3_O_4_ NPs have a very low toxicity and are biocompatible [7,8].

There is fundamental interest in the packaging of multiple magnetic nanocrystals in a well-defined host material, along with the accompanying impact on physical properties [9–13]. One appealing option involves high surface area elemental silicon (Si) for this purpose, in the form of porous Si NPs [14,15] or silicon nanotubes (Si NTs) of a well-defined size and uniform structures. The NT length, along with the outer and inner diameter of the Si NTs, is in principle broadly tunable, with a wall thickness-dependent aqueous dissolution behaviour [16].

The characteristic quality of an MRI contrast agent is typically measured by the parameters of relaxivity *r*_1_ or *r*_2_, which describe the ability of a given contrast agent to shorten the *T*_1_ or *T*_2_ relaxation time of water [17]. The most popular class of contrast agents in current use involves Gd-complex based *T*_1_ MRI contrast agents, with extensive development of nanoparticulate *T*_1_ contrast agents containing Gd^3+^ or Mn^2+^ ions intensively pursued in recent years [18]. However, toxicity problems still persist in these NP-based *T*_1_ contrast agents, thereby motivating additional studies towards the establishment of new alternatives. Of possible choices, Fe_3_O_4_ offers a superior biocompatibility relative to the above gadolinium-based materials [19]. Fe_3_O_4_ MRI contrast agents are considered a negative contrast agent (or *r*_2_ weighted), with the possible enhancement often determined by several factors such as: (i) NP size; (ii) composition; (iii) surface coating; and (iv) synergistic magnetic effects arising from the presence of multiple superparamagnetic Fe_3_O_4_ NP centres in a relatively small volume [20].

For MRI applications, Fe_3_O_4_ NPs have been functionalized with different surface moieties to increase solubility [21]. The local concentration of Fe_3_O_4_ in solution has also been increased by the assembly of other structures such as Fe_3_O_4_ nanorods and clusters of individual Fe_3_O_4_ NPs [22,23]. However, these clusters often lack uniformity and a well-defined three-dimensional structure. There is one report of Fe_3_O_4_ NPs formed in carbon NTs [24]. Since the Fe_3_O_4_ NPs are formed *in situ* inside the NTs, the Fe_3_O_4_ nanostructures do not have a uniform particle size distribution [25]. To our knowledge, there are no reports of Si NTs loaded with Fe_3_O_4_ NPs for MRI applications.

In this article, we describe loading processes for incorporating Fe_3_O_4_ NPs into SiNTs, routes to surface modification of such loaded NTs for proper solubility, and the relaxivity properties of these Fe_3_O_4_ NP-loaded Si NTs. We selected Si NTs of well-defined thickness (40 nm and 70 nm) that will not degrade during the timescale of the relaxivity measurements. To examine the role of Fe_3_O_4_ nanocrystal size on loading and associated properties, we use NPs of both 5 nm and 8 nm diameter for this purpose. These experiments complement our earlier experiments involving investigations of the fundamental magnetic properties (blocking temperature, temperature-dependent coercivity) of new materials potentially useful as an MRI contrast agent based on Fe_3_O_4_ NPs loaded into silicon nanostructures [26].

## 2. Material and methods

### 2.1. Iron oxide nanoparticles

The Fe_3_O_4_ NPs used here were fabricated using a well-known route involving high temperature decomposition of a suitable molecular iron precursor. Further details regarding the fabrication process of these NPs can be found in previous publications [27].

### 2.2. Silicon nanotubes

Si NTs were fabricated by a sacrificial template method reported previously by our research group [16]. In general terms, it involves the initial formation of ZnO nanowire array (NWA) templates on a substrate (such as silicon wafers or F-doped tin oxide (FTO) glass), followed by Si deposition (540°C for 40 nm shell thickness Si NTs, 580°C for 70 nm shell thickness) and subsequent template removal by an NH_3_/HCl etch under a helium atmosphere at 400°C. ZnO NWA templates were prepared on a given substrate (FTO or Si) that were previously seeded with ZnO nanocrystals (according to a previously described procedure) by placing in a mixture (1 : 1 v : v) of 0.03 M Zn(NO_3_)_2_ and 0.03 M hexamethylenetetramine at 92°C for 9 h. Polyethylenimine (100 µl, branched, low molecular weight, Aldrich) was added into 100 ml of ZnO growth solution. A given ZnO NWA sample was inserted into a quartz tube reactor and Si deposition on the ZnO NWA was achieved through the use of diluted silane (20 sccm, 0.5% in He) mixed with He carrier gas (200 sccm) that was passed through a furnace. These Si-coated ZnO NW samples were then placed in another quartz reactor and heated to 450°C; NH_4_Cl was loaded in an alumina boat located upstream and heated to 350°C. The gaseous etchant was transported via He gas downstream (170 sccm) to the furnace for 1 h for removal of the ZnO NWA template.

### 2.3. Loading of Fe_3_O_4_ NPs into silicon nanotubes

**Figure 1.**
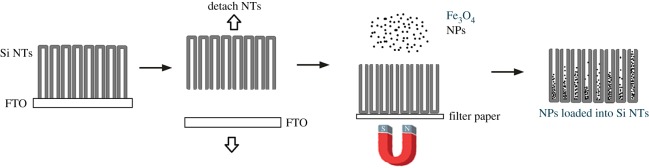
Loading process of Fe_3_O_4_ NPs into silicon nanotubes (Si NTs). Si NT arrays are physically detached from their substrate; the film is then inverted and exposed to a solution of Fe_3_O_4_ NPs with a bar magnet underneath.

The process for loading Fe_3_O_4_ NPs into these NTs is illustrated in figure 1. This is readily achieved by initial physical removal of the Si NT film from the underlying substrate (such as FTO glass) and placing it face down on top of an Nd magnet with a piece of filter paper in between. Fe_3_O_4_ NPs (oleic acid terminated, hexane solution) at a concentration of 7 mg ml^−1^ are added dropwise, followed by rinsing the infiltrated sample with acetone several times and allowed to air dry.

### 2.4. Ferrozine assay

A Ferrozine assay was used to determine the concentration of iron [28]. This assay consists of taking a 400 µl solution containing a known mass of Si NTs loaded with Fe_3_O_4_ NPs. This solution is then mixed with 400 µl of 12 M HCl and allowed to sit for 1 h, this solution is mixed with 400 µl of 12 M NaOH to neutralize the solution, followed by addition of 96 µl of 2.8 M HONH_2_ in 4 M HCl, followed by a waiting period of 1 h. Ammonium acetate (40 µl 10 M) is then added, followed by 240 µl of 300 mM of ferrozine dissolved in 0.1 M of ammonium acetate. These solutions are allowed to sit overnight and the absorbance of a given solution at 562 nm is recorded.

### 2.5. APTES and PEG-diacid (600) functionalization

Si NTs were immersed in 1% (3-aminopropyl)triethoxysilane (APTES) solution for 4 h in acetone, followed by rinsing with deionized (DI) water. Poly(ethylene glycol) diacid 600 (PEG-diacid 600) functionalization is achieved using APTES-functionalized Si NTs. This procedure involves the preparation of a solution of PEG-diacid (600) (3.3 mmol), 1-ethyl-3-(3-dimethylaminopropyl) carbodiimide (1 mmol) and N-hydroxysuccinimide (1 mmol) in 2 ml of DI water. This mixture is stirred for 15 min. Then APTES-Si NTs was added (0.8 mg) and stirred for 3 h. The product was dialysed against DI water by changing the DI water several times. The samples were stored in solution at room temperature for further use.

## 3. Results and discussion

### 3.1. Structural and morphological characterization

**Figure 2.**
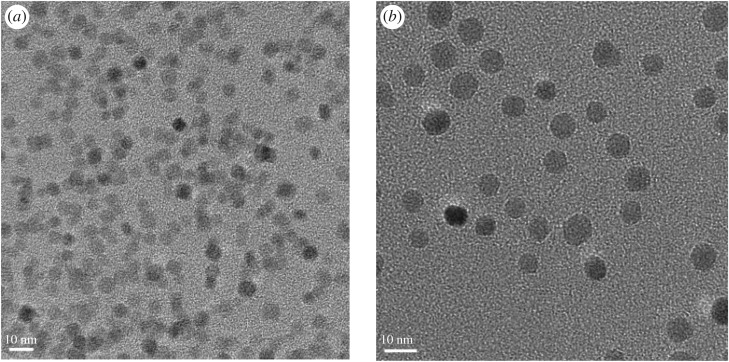
TEM images of Fe_3_O_4_ NPs of (*a*) 5 nm average diameter and (*b*) 8 nm average diameter.

Transmission electron microscopy (TEM) analysis (JEOL JEM-2100) confirms that the Fe_3_O_4_ NPs used in these experiments are uniformly spherical (figure 2) and have narrow size distributions of 5.10 ± 0.98 nm and 8.15 ± 1.76 nm (for associated histograms see the electronic supplementary material, figure S1). High-resolution TEM analysis confirms that each particle consists of well-oriented single domains with the measured distance between two adjacent lattice fringes of a value of 0.258 nm (electronic supplementary material, figure S2), close to the reported value of 0.253 nm (corresponding to the lattice spacing associated with the (311) planes of Fe_3_O_4_), thereby supporting the presence of the magnetite structure.

**Figure 3.**
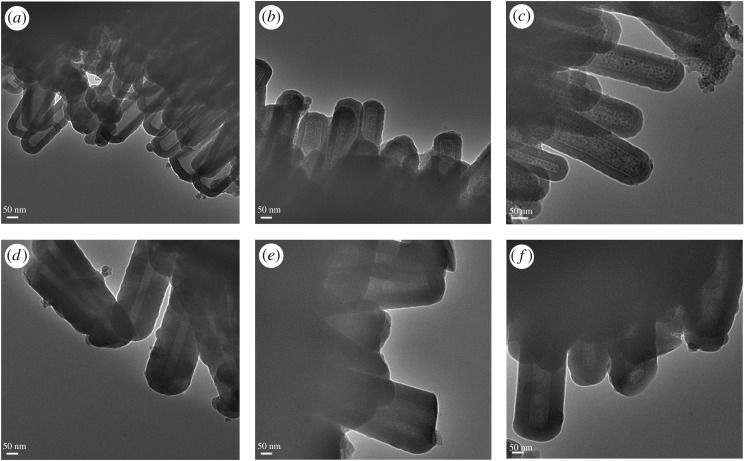
(*a–c*) TEM imaging of 40 nm Si NTs: (*a*) empty Si NTs; (*b*) Si NTs loaded with 5 nm Fe_3_O_4_; (*c*) Si NTs loaded with 8 nm Fe_3_O_4_. (*d–f*) TEM of 70 nm Si NTs: (*d*) empty Si NTs; (*e*) Si NTs loaded with 5 nm Fe_3_O_4_; (*f*) Si NTs loaded with 8 nm Fe_3_O_4_.

Si NTs offer a uniform wall structure and relatively large interior loading capacity, with a separation distance of magnetic NPs between tube interiors that is effectively two times the wall thickness of a given type of Si NT. TEM also confirms the successful loading of Fe_3_O_4_ NPS into the Si NTs. Figure 3*a* shows empty Si NTs with a wall thickness of 40 nm; figure 3*b*,*c* shows these Si NTs loaded with Fe_3_O_4_ NPs of 5 nm and 8 nm, respectively. In figure 3*d*, empty Si NTs with a wall thickness of 70 nm are presented, with figure 3*e*,*f* presenting these Si NTs loaded with 5 nm and 8 nm Fe_3_O_4_ NPs, respectively. We observe a uniform filling of Fe_3_O_4_ NPs inside a given Si NT. No Fe_3_O_4_ NPs are observed outside these Si NTs, meaning that the washing process was successful, with no significant amount of Fe_3_O_4_NPs removed from inside the Si NTs.

**Figure 4.**
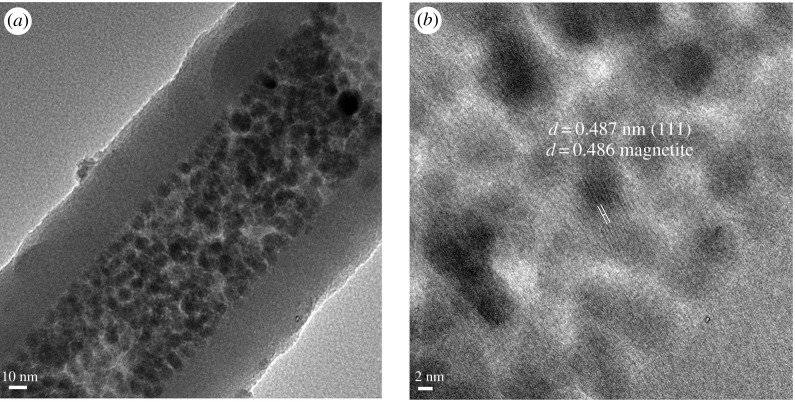
(*a*) Fe_3_O_4_ NPs (8 nm average diameter) loaded into 40 nm wall thick Si NTs; (*b*) high-resolution TEM imaging of this sample, showing {111} spacings associated with the magnetite phase.

**Figure 5.**
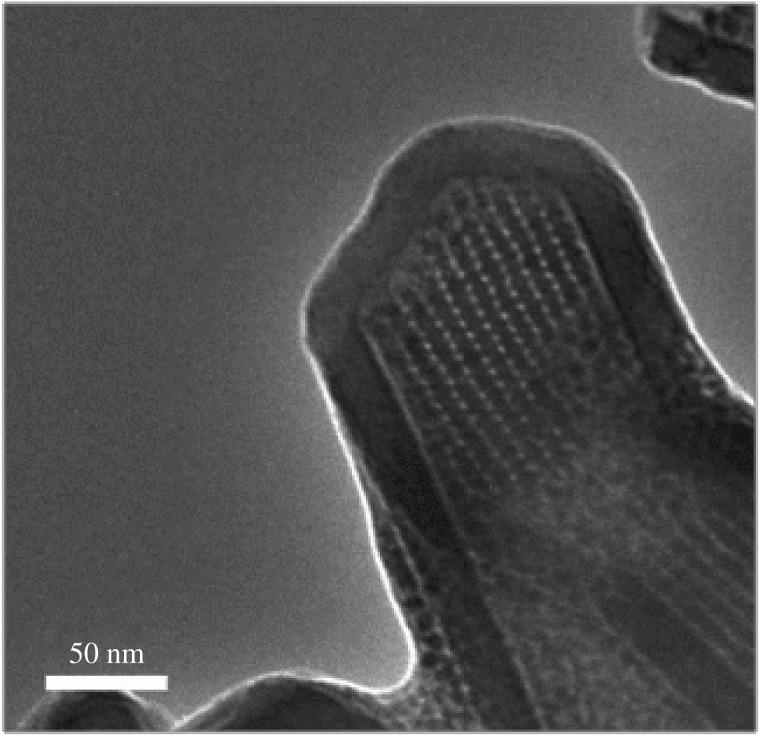
Si NT loaded with Fe_3_O_4_ NPs, illustrating the closest packing of the nanocrystals inside the tube.

It should also be pointed out that under high magnification conditions, lattice planes of Fe_3_O_4_ NPs can be imaged inside the NTs, confirming the presence of the magnetite structures at this location. The lattice spacing of the plane of the magnetite phase can be readily confirmed (figure 4*a*). Interestingly, in selected NTs a closest packed arrangement of Fe_3_O_4_ NPs can also be observed (figure 5).

### 3.2. Magnetic characterization

**Figure 6.**
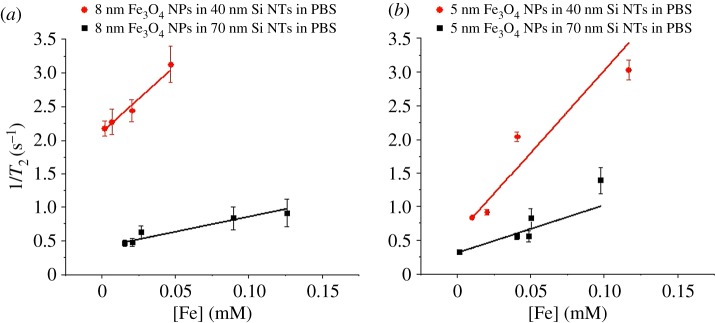
1/*T*_2_ versus [Fe] of Si NTs loaded with: (*a*) 8 nm Fe_3_O_4_ NPs and (*b*) 5 nm Fe_3_O_4_ NPs.

To evaluate the potential use of this new material as an MRI contrast agent, we measured the *T*_1_ and *T*_2_ values of a given sample using a relaxometer (Bruker Minispec mq60) at 1.41 T. *T*_1_ and *T*_2_ values were recorded in phosphate buffered saline (PBS) at 37°C. A linear dependence is observed between the inverse proton relaxation times and the iron concentration according to the following equation:

3.1 $$\frac{1}{T_{i,\mathrm{obs}}}=\frac{1}{T_{i,0}}+r_{i}\;[\text{Fe}],$$

where 1/*T_i_*_,obs_ (*i* = 1, 2) is the inverse relaxation time measured experimentally in the presence of the magnetic nanomaterial, 1/*T_i_*_,0_ is the inverse relaxation time of pure water in the absence of the contrast agent, [Fe] is the iron concentration in the contrast agent and *r_i_* is the longitudinal (*i* = 1) or transverse (*i* = 2) relaxivity (i.e. proton relaxation rate enhancement per mM Fe cation concentration) [29]. Representative plots are shown in figure 6 for 1/*T*_2_ versus iron concentration for all Fe_3_O_4_ NPs housed within Si NTs. Similar plots were undertaken for 1/*T*_1_ (see the electronic supplementary material, figure S3). From linear fits of the plots, *r*_1_ and *r*_2_ can be calculated from the slopes. These *r*_1_ and *r*_2_ values are shown in table 1, as well as the corresponding *r*_2_/*r*_1_ values. As expected, *r*_2_/*r*_1_ > 2 is consistent with the classification of all Fe_3_O_4_—containing structures as a negative contrast agent. This is also true from the observation that *r*_1_ values are observed here in the range of 0.05–0.35 mM^−1^ s^−1^.

**Table 1.** *r*_1_, *r*_2_ and *r*_2_/*r*_1_ values associated with Fe_3_O_4_ NPs loaded into different Si NTs.

5 nm Fe_3_O_4_ in:	*r*_1_ (mM^−1^ s^−1^)	*r*_2_ (mM^−1^ s^−1^)	*r*_2_/*r*_1_	8 nm Fe_3_O_4_ in:	*r*_1_ (mM^−1^ s^−1^)	*r*_2_ (mM^−1^ s^−1^)	*r*_2_/*r*_1_
40 nm wall Si NTs	0.15	20.59	138.16	40 nm wall Si NTs	0.62	21.05	35.04
70 nm wall Si NTs	0.37	11.31	30.32	70 nm wall Si NTs	0.31	3.96	12.93

Some trends emerge from a simple analysis of the measured *r*_2_ values. For all Fe_3_O_4_ NPs loaded into Si NTs possessing 40 nm wall thickness, *r*_2_ is apparently insensitive to particle size, with a value of approximately 21 mM^−1^ s^−1^. Also, for a given Fe_3_O_4_ NP size, we observe the fact that *r*_2_ drops a minimum of 50% with increasing wall thickness (from 40 to 70 nm).

To interpret the above trends, it is useful to examine the following size-dependent expression for *T*_2_ [30]:

3.2 $$\frac{1}{T_{2}}=\left(\frac{256\pi^{2}\gamma^{2}}{405}\right)\;\frac{V^{*}M_{S}^{2}a^{2}}{D(1+(L/a))},$$

where *γ*, gyromagnetic ratio; V*, volume fraction; *a* radius of Fe_3_O_4_ core; *D* diffusivity of water molecules; *L*, thickness of an impermeable surface coating.

By looking at the above results, it appears that for samples in PBS, the changes in *T*_2_ values are dictated by thickness of surface coating. *T*_2_ relaxivity decreases as the Si NTs wall thickness increases, as presumably thicker Si NTs are less permeable to aqueous diffusion.

It is also informative to compare these *r*_1_ and *r*_2_ values with representative data from the existing literature. For example, Zhou obtained an *r*_1_ value for 5 nm Fe_3_O_4_-coated with dimercaptosuccinic acid (DMSA) of 6.21 mM^−1^ s^−1^, an *r*_2_ of 39.53 mM^−1^ s^−1^ and *r*_2_/*r*_1_ of 6.58 (at 1.5 T) [31]. Huang *et al.* reported that 5 nm Fe_3_O_4_ coated with sodium tartrate shows an *r*_1_ of 4.3 mM^−1^ s^−1^, an *r*_2_ of 23 mM^−1^ s^−1^ and *r*_2_/*r*_1_ of 5.3 [32]. Mamor and co-workers report that 8 nm Fe_3_O_4_ NPs coated with citric acid possesses an *r*_1_ of 28.8 mM^−1^ s^−1^, an *r*_2_ of 54.4 mM^−1^ s^−1^ and *r*_2_/*r*_1_ of 1.89 [33]. It is important to consider that in our system, Fe_3_O_4_ NPs are not widely dispersed in the medium. Rather, all Fe_3_O_4_ NPs are concentrated in a one-dimensional construct inside a given Si NT, but these Si NTs are well dispersed in the medium as a result of PEG (diacid) surface functionalization. Based on the data presented here, the very low values obtained for *r*_1_ (in general, less than 1 mM^−1^ s^−1^) suggests that protons in a given medium do not have facile access to the surface of the encapsulated Fe_3_O_4_ NPs.

For this reason, it is perhaps more appropriate to compare our system with relaxometry data from Fe_3_O_4_ clusters comprised aggregates of similar sizes of Fe_3_O_4_ NPs previously reported [34]. Resovist^®^, commercially available Fe_3_O_4_ NPs clusters coated with carboxydextran, have an overall diameter of 60 nm with an individual particle size of 4.6 nm; this cluster has an *r*_1_ of 10.9 mM^−1^ s^−1^, an *r*_2_ of 190 mM^−1^ s^−1^ and *r*_2_/*r*_1_ of 17.4 [35]. Qin *et al.* reported data for a cluster size of 71 nm with an individual particle size of 10.1 nm; this cluster is coated with Pluronic F127. These workers obtained an *r*_1_ of 0.31 mM^−1^ s^−1^, an *r*_2_ of 71 mM^−1^ s^−1^ and *r*_2_/*r*_1_ of 229 [36]. Tilborg and co-workers reported a cluster size of 62.4 nm coated with PEG2000 and an individual particle size of 9 nm. An *r*_1_ of 0.62 mM^−1^ s^−1^, *r*_2_ of 402.48 mM^−1^ s^−1^ and *r*_2_/*r*_1_ of 647 was obtained for this system [37]. However, the extremely high value of the latter system is probably influenced by the fact that the experiments were made at 9 T versus the 1.5 T used in our experiments. It should also be noted that those clusters are spherically shaped aggregates of small spherical particles, in contrast to the rod-like structure of our NTs. However, in general, the *r*_2_/*r*_1_ values we obtained are in the range of clusters previously reported. Most importantly, however, it is significant to note the ability of the NT wall thickness to mediate the relaxivity values (with regard to *T*_2_), a topic warranting further investigation and ideal expansion of the tunability of these parameters as a function of template geometry and surface chemistry.

## 4. Conclusion

This work describes a straightforward process for the incorporation of superparamagnetic Fe_3_O_4_ NPs inside Si NTs, along with a necessary surface modification strategy for PEG functionalization. While the observed *r*_2_ values of these Fe_3_O_4_/Si NT composites are consistent with the clustering of Fe_3_O_4_ NPs in the NT interior, the ability to achieve ordered closest packed nanocrystal arrays is a unique attribute of this system. Further detailed evaluation of the ability of these materials to act as a negative contrast agent *in vivo* remain.

## Supplementary Material

Figures S1 - S3

## Data accessibility

Datasets are deposited at Dryad: (http://dx.doi.org/10.5061/dryad.k2vh732) [38].
