# Supplementary material for: New MRI contrast agents based on silicon nanotubes loaded with superparamagnetic iron oxide nanoparticles
Source: R Soc Open Sci. 2018 Aug 1;5(8):180697. doi: 10.1098/rsos.180697 (PMC6124042; doi:10.1098/rsos.180697)
Supplement: Figures S1 - S3 [file rsos180697supp1.pdf]

Supporting information

New MRI contrast agents based on silicon nanotubes loaded with superparamagnetic iron oxide nanoparticles

Roberto Gonzalez-Rodriguez,[a] Petra Granitzer,[b] Klemens Rumpf,[b] and Jeffery L. Coffer[a]*

[a.] Department of Chemistry, Texas Christian University, Fort Worth, Texas. USA. 76129. Tel: 817-257-5355. Email j.coffer@tcu.edu
[b.] Institute of Physics, Karl-Franzens-University Graz, Universitaetsplatz 5, A-8010 Graz, Austria.

A)

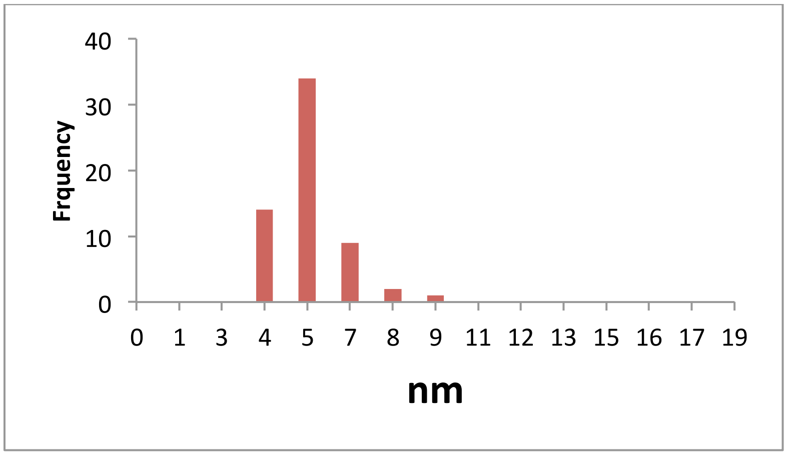

Mean	5.1 nm
SD	0.981 nm

B)

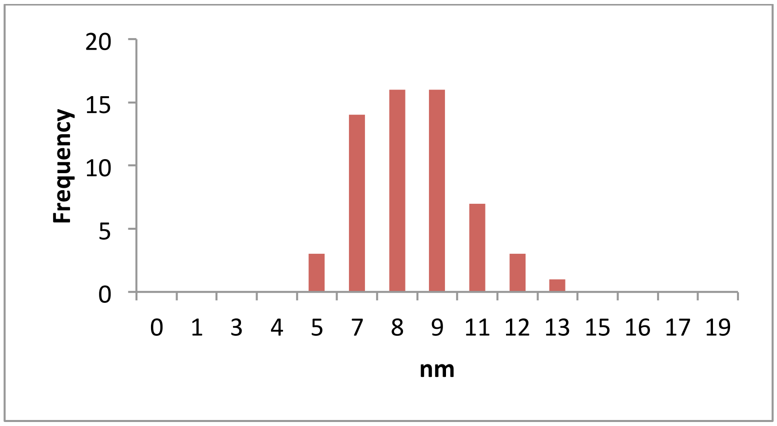

Mean	8.159 nm
SD	1.763 nm

Figure S1. Iron oxide nanoparticles size distribution. A) 5 nm average diameter Fe_3O_4. B) 8 nm average diameter Fe_3O_4.

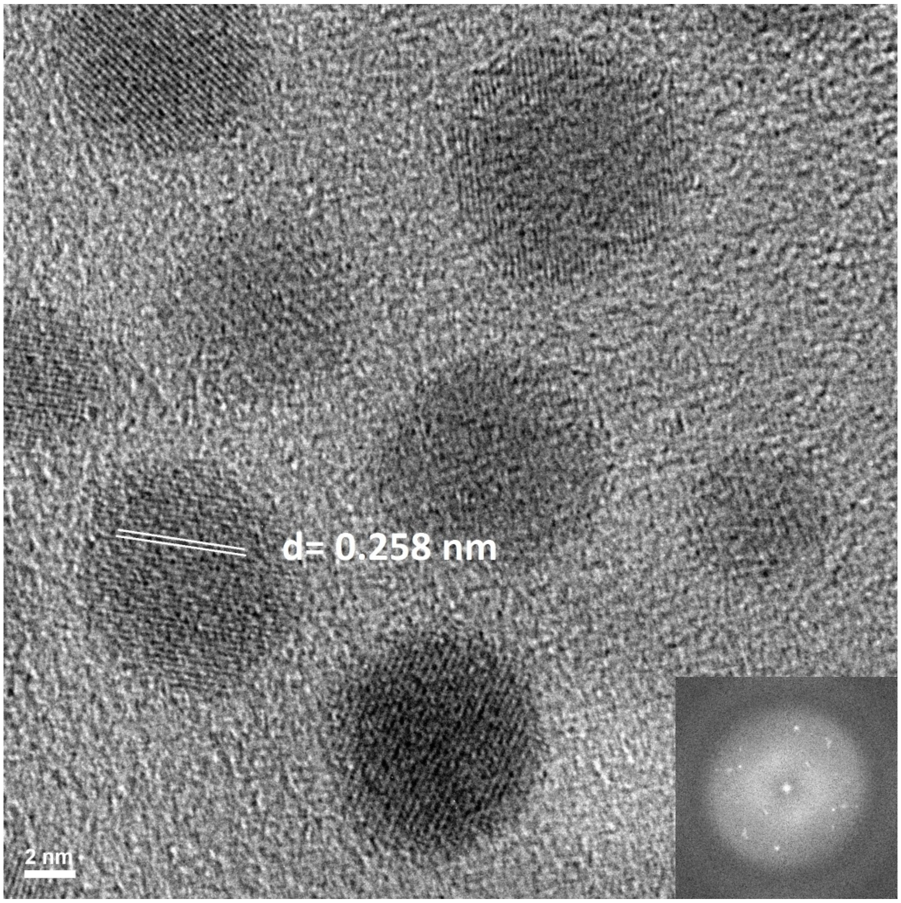

Figure S2. HRTEM of Fe_3O_4 NPs and associated FFT. Scale bar = 2 nm.

A)

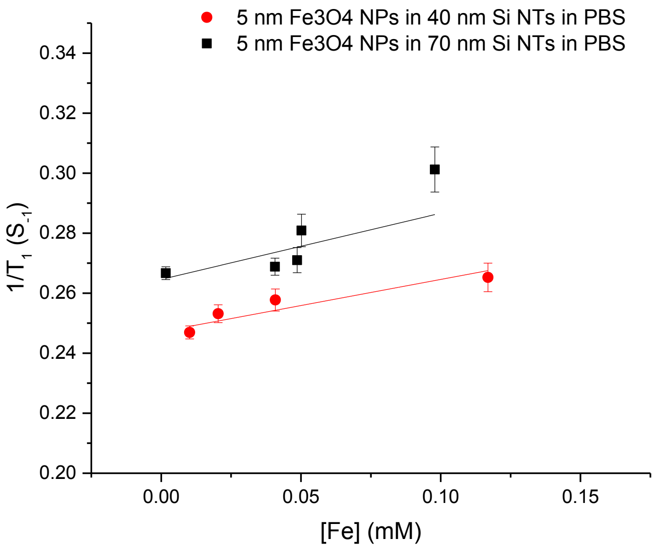

B)

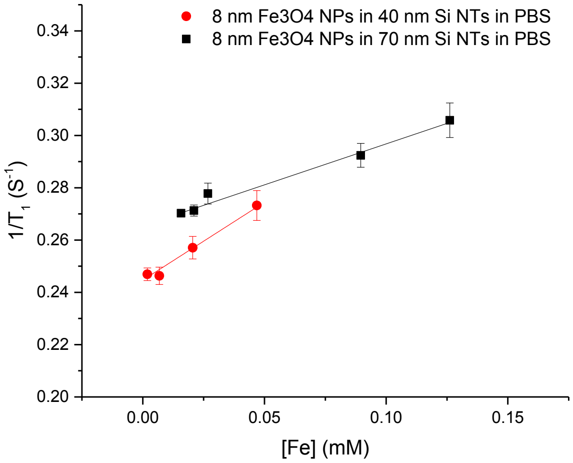

Figure S3. Iron concentration versus $1/T_1$ for Fe_3O_4 NPs in 40 nm and 70 nm wall thickness Si NTs: a) SiNTs loaded with 5 nm Fe_3O_4; b) SiNTs loaded with 8 nm Fe_3O_4.
